# EPA position paper

**DOI:** 10.1192/j.eurpsy.2025.66

**Published:** 2025-08-26

**Authors:** M. Destoop

**Affiliations:** 1 CAPRI, Antwerp University; 2 Multiversum, Antwerp, Belgium

## Abstract

**Abstract:**

The EPA acknowledges both the therapeutic potential of psychedelic substances and the challenges for both research and clinical implementation. Steps need to be taken towards a well-balanced policy based upon sound scientific evidence and research, aiming at safe, ethical responsible integration of psychedelic therapy available for all patients who can potentially benefit. In the EPA policy paper the importance of the psychosocial components of the treatment as well as the ethical and professional aspects playing a role in real-world implementation, are highlighted. Four recommendations are formulated for further research and clinical implementation.

**Disclosure of Interest:**

None Declared

